# Carcinoma of the Colon; Its Pathology and Symptoms*Read before Houston Pathological Society, April, 1914.

**Published:** 1915-07

**Authors:** Claude C. Cody


### TEXAS MEDICAL JOURNAL

Dr. F. E. Daniel, Founder. Established July, 1885

MRS. F. E. DANIEL, - Publisher and Managing Editor

Published Monthly.—Subscription, $1.00 a Year.

VOL. XXXI AUSTIN, JULY, 1915 NO. 1

The publisher is not responsible for views of contributors

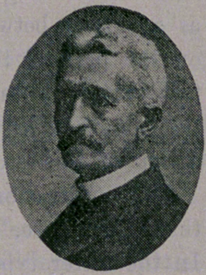

### Original Articles.

### Carcinoma of the Colon; Its Pathology and Symptoms.*

BY CLAUDE C. CODY, M. A., M. D.

The pathology of carcinoma of the colon conforms in the main to that of cancer in other regions; while the symptoms are due to the structural alterations of the large intestine consequent upon the growth of the tumor, and only rarely to the neoplasm itself. The symptoms and pathology bear to each other such a dependent relation that when one is known it is usually possible to foretell the other. This relation is of more than academic interest, for by it the treatment is determined by differentiating the operable from the inoperable cases.

A few of the etiological factors have peculiarities worth mentioning. The *cancer age* can be recognized, for those presenting the disease range in years from three to eighty-four; and about 15 per cent appear in individuals below thirty. No other lesion is constantly associated with it. Males are affected twice as often as females. It is rarely seen among negroes. The cecum and ascending colon are most frequently the site of the tumor.

The mode of growth is from a primary cancerous degeneration in the mucosa, which enlarges by expansion or infiltration toward the periphery. The *expansil* type of neoplasm grows by the development of a carcinomatous parenchyma—the medullary form; or by the development of a connective tissue stroma—the scirrhus form; or by a combination of these—the simplex. The neoplasm

*Read before Houston Pathological Society, April, 1914.

grows as a compact mass with but little cancerous outcroppings and assumes a globular form. A layer of connective tissue acts as a barrier between the sound and malignant cells, in an effort on the part of the organism to isolate the neoplasm; and from this protective barrier strands of connective tissue extend through the tumor. The formation of adhesions, therefore, is but a modification of this protective process.

The infiltration method of enlargement is far more frequent than the above, and is either *interglandular* or *intraglandular.* In the latter type, the origin of the neoplasm is in an interruption of the continuity of the mucous membrane, and it grows by an infiltration through the mucosa or submucosa. The *interglandular* form arises in practically the same manner but spreads almost exclusively by the lymph vessels. According to the anatomy of the lymph channels, the cancer cells may grow horizontally along the periglandular and subglandular groups of lymph channels medial to the muscularis mucosa into the rich submucous groups. When the filtration is horizontal, the colon is converted into a rigid tube; when peripheral, the large intestine is surrounded by a relatively narrow band. Consequently the macroscopical forms of carcinoma are classified as tubular, annular, and in the expansil type, globular; but, of course, these present frequent combinations. The malignant cells constantly tend to reach the subperitoneal group, where the irritative reaction of connective tissue causes adhesions. These indicate the extent of carcinomatous infiltration of the subperitoneal lymph channels, and in conjunction with the size of the neoplasm serve as an index of its malignancy. In addition to their prognostic value, adhesions are important clinically from a mechanical standpoint, for they produce volvulus, kinking of the bowel, bizarre malformations of the intestines, and with perforation cause pathological anastomosis between segments of the colon.

The size of the neoplasm varies from a narrow band 3 centimeters broad to a mass with the dimensions of 18x36 centimeters. The consistency is hard and elastic; the surface is nodular, but rarely smooth. However, in the types presenting degeneration, the tumor may be soft and semi-fluctuant, and the surface glassy. The edges of the neoplasm abutting the mucous membrane are wiry, raised, hard, and well-defined. Ulceration of the mucosa is present in about one-half of the cases. The ulcer is usually superficial; but it frequently extends through the wall of the colon, causing abscesses, local or general peritonitis. In addition to perforation, peritonitis is caused by cancerous extension along the subperitoneal lymphatics with the formation of abscess and by metastases to the peritoneum.

The annular and tubular forms of the neoplasm are very prone to cause an obstruction to the flow of intestinal contents. The growth of the tumor, the proliferation of the protective layer of connective tissue, and particularly the edema of the mucous membrane, produce a constantly increasing construction of the large intestine with a consequent deleterious effect upon the colonic walls. The colon distal to the neoplasm differs but little from normal; in fact, it may be shrunken. The changes in the structure of the proximal wall are due to the progressive stenosis by the neoplasm and to the stasis of the feces. The hypertrophy of the muscular wall is dependent upon the degree of stenosis; the smaller the opening, the greater the hypertrophy. The narrowing of the lumen may continue untii even the hypertrophied muscles are unable to force the feces past the obstruction. As a result, there are stagnation of the intestinal contents, auto-intoxication, and edematous, friable, and, in extreme cases, paralyzed colonic walls. If this condition continues unrelieved, even the hypertrophied muscles may become insufficient, and acute dilatation of the colon ensues with failure in compensation of the intestinal muscles, or retro-peristalsis. This is evidenced clinically by the sudden appearance of a large tumefaction, fecal vomiting, collapse; and, if not alleviated, speedy death. On the other hand, chronic dilatation presents no such violent picture, and may exist for some time with no untoward results.

The relative rarity of metastasis is remarked by all recent writers, some going so far as to say it practically never occurs. This is due, primarily, to the fact that the type of carcinoma is not so malignant, and, secondarily, to the poverty in the supply of lymphatics to the large intestine, as pointed out by Mayo. However, the malignant type of cancer shows metastases in 100 per cent of the cases. The paths taken by the metastases are fairly regular. When the neoplasm is in the cecum metastases may appear in the adjacent portion of the large intestine, in the stomach, in the supraclavicular glands; but not in the liver or pancreas. The tumors of the hepatic flexure may show them in the adjacent portions of the colon, mesocolon, small intestine, in the liver, in the stomach, and in the supraclavicular glands. In neoplasms of the sigmoid they may appear in the adnexa, adjacent mesocolon and section of the colon, and particularly in the liver.

The pathology common to all cancers of the large intestine have been presented above, and the peculiarities of each variety will now be considered. Petersen and Colmers propose the following classification:

I.

Adeno carcinoma.
Adeno carcinoma gelatinosum.
Adeno carcinoma microcysticum.

II.

Carcinoma solidum alveolare.
Carcinoma solidum gelatinosum.
Carcinoma solidum diffusum.

*Adeno Carcinoma.*—The size varies from that of a walnut to that of a muskmelon, and the form is usually tubular. The consistency is hard, and the surface is nodular. It may be divided histologically into adeno carcinoma cylindrico-cellulare, and adeno carcinoma cubo-cellulare; the former occurs twice as frequently as the latter. The neoplasm of the cylindrico-cellulare type is separated from the neighboring cells by a well-developed connective tissue strata; and from this protective barrier strands extend through the tumor developing into stroma. The form of the glands may be changed, or their number greatly increased; but in either case the epithelium differs but little from normal. The glandular epithelium begins its carcinomatous proliferation in the fundus with the formation of many rows of normal or slightly modified cells. The tendency of the glandular epithelium to the formation of adenoma is not lost either in extensions along the colonic wall, or in metastases to distant parts. The proliferation of epithelium may continue until the glandular character of the neoplasm is metamorphosed into solid cylinders. When this occurs, the cubo-cellulare appears with the glandular type of cell changed from its normal or slightly modified cylindrical appearance to a flat, polygonal or cuboidal cell, with a large, deeply staining nucleus; in other words, a type of cell closely resembling that seen in epithelioma cubo-cellulare of the skin. The protective barrier of connective tissue is slight or absent, the extension of the growth is very free, cells vary greatly in form, and mitotic figures abound. The differential characteristics between the two classes are the change in type of cell; the relative or absolute absence of the connective tissue barrier in the cubo-cellulare; the method of extension and metastases, the cylindrico-cellulare by adenomata, and the cubo-cellulare by solid cylinders; the tendency of the cubo-cellulare to form metastasis; and the invariable death of patients affected with the cubo-cellulare.

*Adeno Carcinoma Gelatinosum.*—Adeno carcinoma gelatinosum is a mucoid degeneration of the adeno carcinoma. The degeneration may appear at any stage of the neoplasm's growth. Its size is usually larger than the adeno carcinoma, and its form is usually globular. It is readily distinguished from the other forms by the vesicles of clear, viscid, material shimmering through the tissues, and by the peculiar palpatory consistency. Perforation is rare. The histology of this type does not differ from the preceding; as in the extension of growth and in the metastases the carcinomatous cells are seen preceding the gelatinous degeneration. There is little tendency for the tumor to extend by lymphatics. When it breaks through the peritoneum, it is disseminated widely by extension along the serosa, or forms metastasis by the currents of free lymph in the peritoneal cavity carrying the cells to distant part. The early growths of either extension of metastasis appear only on the serosa, and the invasion of the viscus is from this point and not from the lymphatic sinuses.

*Adeno Carcinoma Microcysticum.*—The characteristic of this type is enormous number of small glands; otherwise it is the same as adeno carcinoma.

II.

Carcinoma solidum is the end result of that metamorphosis from the adenomatous structures of glandular epithelium to solid areas of flat polygonal, and oval cells. This type is more malignant than the adeno carcinoma.

Carcinoma solidum alveolare and carcinoma solidum diffusum differ only in the form of the tumor, mode of extension, and in the arrangement of the cancerous cells. The carcinoma alveolare has a tubular form, and extends by infiltrative processes; the carcinoma diffusum is globular, and expansil in type. The arrangement of the cancerous cells in the carcinoma alveolare is in alveoli, separated by connective tissue stroma, but it is made up wholly of carcinomatous parenchyma, or medullary in type. Carcinoma solidum gelatinosum is derived from carcinoma alveolare and carcinoma diffusum in the same way that adeno gelatinosum is degeneration of adeno carcinoma. It has the same pathological concomitants as described above for the other two types, except its extension and metastases are after the serosa has been penetrated.

SYMPTOMS.

From the above pathological description, it is seen that there are two well-marked varieties of carcinoma. One of these is the type characterized by an abundant growth of connective tissue in such a way that it produces a mechanical blocking of the flow of intestinal contents; the other type relies on its malignancy for its clinical manifestations and evidences itself more as a toxemia. The first class we denote obstructive; and, the second, non-obstructive.

*Obstructive Group.*—The obstructive group includes all cases of adeno carcinoma cylindrico-cellulare, adeno carcinoma gelatinosum, adeno carcinoma microcysticum; and very rarely those of adeno carcinoma cubo-cellulare and carcinoma solidum gelatinosum which are very large.

The onset is commonly insidious, with the first symptom as pain, next constipation and later nausea and vomiting. The characteristic feature of this pain is the occurrence of repeated attacks, with the intervals becoming shorter the more mature the neoplasm. General abdominal colic is present in one-third of the cases; while the pain is localized in two-thirds, and is referred to as "colicky," "sharp," or "dragging sensations." Little reliance can be placed on the localization of pain as an indication of the neoplasm's site, though it occurs most frequent in the lower right quadrant. Nausea and vomiting is a late symptom and causes but little straining. The character of the vomitus is gastric; rarely fecal. Constipation is more subject to variations than the other two cardinal symptoms, and it does not alternate with diarrhea frequently. The stools are ribbon, or pencil-shaped and scybala abound. Blood and mucus appear in one-third of the cases, and, of course, is indicative of ulcer. There is usually a progressive loss in the patient's weight, which may vary from a few pounds to fifty. An increase in weight just before the appearance of the first symptom is not infrequently noted. The duration of symptoms has no characteristic time-limit, but varies from three weeks to three years.

A palpable tumor is found about half the time. It is quite mobile—except in the cecum, ascending colon, and descending colon; being easily demonstratable at one examination, and found only with difficulty at the next, or vice versa. The tumor is hard and firm, and usually nodular; though the palpation clinically follows the pathological consistency. Tenderness occurs in only one-fourth of the patients. Visible peristalsis is the most characteristic single symptom of this group, and is demonstrated usually in one-half of the cases.

The value of a diurnal variation in temperature is insisted upon by Mayo and Armstrong. This appears in the cases with ulcerated mucosa or abscesses, and, therefore, seems to be more directly due to sepsis than to cancer. Anemia is invariably present, and the leucocytosis is low except in ulceration, perforation or abscess. Examination of the gastric contents is uniformly negative.

The diagnosis rests on the following syndrome: recurrent spasmodic pain, nausea and vomiting, loss in weight, anemia, constipation, asthenia; and when demonstratable, the tumor, peristalsis, and blood in the stools. The X-ray examination is very valuable. Pieces of cancerous tissue are rarely washed out by enema.

The *non-obstructive* group includes only those cases of adeno carcinoma cubo-cellulare and carcinoma solidum. There is no such syndrome of symptoms in this group as in the preceding; nor are those which do appear, so constant here as in the obstructive group. Pain appears in practically every case, and usually intermittent. The relation of the reference of pain to the site of the tumor is more exact in this group than in the other. The character of the pain is a soreness and stiffness of the abdominal wall; and, in the late stages, may be very severe. Nausea and vomiting are absent; and constipation is rare. Loss of weight is present. The duration of symptoms in this malignant group varies from two months to over three years, but, as a rule, is shorter than in the obstructive group.

The tumor is demonstrated in practically every case, and is firm and nodular; but is not so mobile as in the preceding group. Tenderness is usually present, but visible peristalsis is not. The diurnal variation of temperature appears in the cases with ulceration. All cases are anemic.

The diagnosis is based upon intermittent pain, the progressive loss in weight, the anemic and the tumor.

---

There is only one way of weeding out mental and moral delinquency, and that is by stopping the output.—*Medical Summary.*

